# Relationship Between Total Rewards Perceptions and Work Engagement Among Chinese Kindergarten Teachers: Organizational Identification as a Mediator

**DOI:** 10.3389/fpsyg.2021.648729

**Published:** 2021-04-29

**Authors:** Dongying Ji, Li Cui

**Affiliations:** ^1^Faculty of Education, Beijing Normal University, Beijing, China; ^2^College of Education, Huaibei Normal University, Huaibei, China

**Keywords:** total rewards perceptions, kindergarten teachers, organizational identification, work engagement, mediating effect

## Abstract

Kindergarten teachers' engagement in work is influenced by many factors. Total rewards perceptions, as an individual's evaluation of the rewards provided by the organization, may promote work engagement when it can meet their intrinsic and extrinsic work demands. To explore the relationship between kindergarten teachers' total rewards perceptions and work engagement, and the mediating role of organizational identification, a survey was conducted among 1,014 kindergarten teachers applying the Chinese versions of the Total Rewards Perceptions Scale for Kindergarten Teacher, Kindergarten Teacher Organizational Identification Scale, and Kindergarten Teacher Work Engagement Scale. The results showed that kindergarten teachers' total rewards perceptions and its four factors were positively correlated with organizational identification and work engagement. Organizational identification was positively related to work engagement. Organizational identification partially mediated the relationship between total rewards perception and work engagement among kindergarten teachers. We discussed the result of the relationship between total rewards perceptions, organizational identification, and work engagement among Chinese kindergarten teachers.

## Introduction

In the past decades, there has been a growing interest in work engagement as research found that engagement is related to meaningful outcomes (Soares and Mosquera, 2019). Work engagement represents a positive, fulfilling, work-related state of mind that is characterized by vigor (high levels of energy and mental resilience), dedication (work involvement, enthusiasm, and inspiration), and absorption (work concentration and immersion) (Schaufeli et al., 2002). Previous studies have shown that levels of teachers' work engagement have a strong influence over job performance, intention to quit teaching, and academic achievement of their students (Roth et al., 2007; Bakker et al., 2008; Duckworth et al., 2009). The teachers' behavior, belief, and emotional dimension are related to the results obtained by the students (Perera et al., 2018). Kindergarten teachers care for and teach children between the ages of three and six. This requires them to devote more energy to cultivating and supporting children's social, emotional, and academic development (Coplan et al., 2015; Stasio et al., 2020).

Total rewards are considered as the critical way that affects members' motivation to join and stay with the organization, and support organizational effectiveness and members' well-being (Muse et al., 2008; Chiboiwa et al., 2010; Newman and Sheikh, 2012a). Total rewards systems have a greater influence on individuals than the single reward because the total rewards system is more flexible to meet the intrinsic and extrinsic demands of organizational members (Armstrong and Stephens, 2005). Monetary rewards and non-monetary rewards are included in total rewards. Among them, monetary rewards include pay perception, benefits perceptions, etc.; non-monetary rewards include learning and development opportunities, feedback and appreciation for work, etc. (Heneman and Tansky, 2002). Strom et al. (2014) found that engagement levels of organizational members depend on their perceptions of the rewards. Social Exchange Theory also indicates that positive work behaviors and attitude of employees are always directed by work resources (Blau, 1964). From this perspective, when individuals perceive resources from their organization, they feel positive about the organization and its values and are willing to engage in work toward the achievement of the organization's goals (Li and Wu, 2014). A rewarding work environment contributes to employees becoming more engaged in their work (Roberts and Davenport, 2002).

The Conservation of Resource Theory proposed by Hobfoll (1989) has believed that individuals always have the tendency to acquire, occupy, and maintain important resources. Sufficient work resources can enhance employees' work motivation, increase work engagement, and improve work performance (Demerouti and Bakker, 2011; Bakker et al., 2014). Rewards, as a kind of resource that employees can access, significantly affect their work engagement (Gulyani and Sharma, 2018). Previous studies have compared the impact of non-monetary rewards and monetary rewards on work engagement and found that the effect of monetary rewards on work engagement is lower than that of non-monetary rewards (Scott and McMullen, 2010). Rewards represent what the organization can offer its employees (Morgan et al., 2013; Chinyio et al., 2018). Therefore, teachers may be more willing to devote themselves to the work if they perceive a greater amount of benefits (e.g., monetary rewards, non-monetary rewards) offered by the kindergarten.

Organizational identification, as the shared beliefs of members (Stuart and Whetten, 1985), represents employees' sense of belonging to the organization (Ashforth et al., 2008) and delimits a set of cognitive, emotional, and behavioral aspects that are consistent with an identity as a member of the organization (Haslam and Ellemers, 2006). It refers to a connection with the organization, and the expectation to improve the status of organization members (Hogg and Terry, 2000). Organizational identification is significantly affected by total rewards perceptions (Yang and Yang, 2015). Du (2013) indicated that working conditions, rewards, and benefits also have a significant impact on organizational identification. Low organizational identification may be due to the imbalance between effort and rewards in the work environment (Guglielmi et al., 2017). The perception of low rewards from the organization may instill a negative social exchange process so that employees are not encouraged to care about the organization (Cropanzano et al., 2003). From the perspective of social exchange theory, some researchers indicated that individuals' identification with organizations partly stems from individuals' perception of organizational support, and it represents the social exchange between individuals and organizations (Shen, 2007). Some studies have shown that a high level of organizational support perception will prompt individuals to show a higher level of organizational identification (Shen et al., 2014; Edwards and Peccei, 2015; Lam et al., 2016). Organizational identification is an important antecedent of work engagement and has a significant impact on work engagement (Riketta, 2005; Guo and Zhang, 2016). Van Dick et al. (2004) explained that organizational identification can improve employees' job satisfaction, and higher job satisfaction may also increase employees' emotional and cognitive involvement in work. Organizational identification not only is influenced by the perception of total rewards but also significantly influences work engagement.

Little research examined organizational identification of kindergarten teachers and the mechanism through which work resources (total rewards components) affect kindergarten teacher work attitude (i.e., teacher work engagement). This study expects to expand the research on organizational identification and explore the mediating effect of organizational identification with Chinese kindergarten teachers as the object. Thus, the current study aims to examine the relationship between total rewards perceptions and work engagement among kindergarten teachers, surveying the mediating effect of organizational identification in the relationship between total rewards perceptions and work engagement among Chinese kindergarten teachers. Accordingly, the main hypothesis of this study is as follows:

Hypothesis 1: Correlations between total rewards perceptions, organizational identification, and work engagement among kindergarten teachers are positive and significant.
Hypothesis 2: Organizational identification mediates the relations between total rewards perceptions and work engagement among kindergarten teachers.

**Figure 1:**
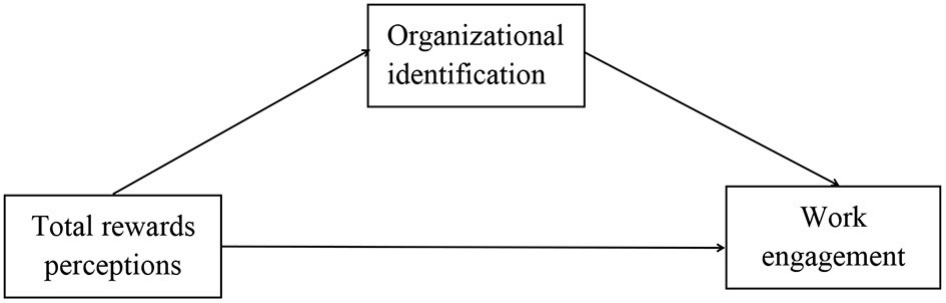
Hypothesized model.

The proposed integrated model is presented in Figure 1.

## Methods

### Participants

All participants were kindergarten professional teachers, excluding the nurses. In this study, a convenient sampling method was adopted to manage the survey and collect large sample data (Axinn and Pearce, 2006). After obtaining consent from the kindergarten principal, the investigators enter the kindergarten to distribute and return questionnaires in 2019. The teachers who participated in this survey were promised that their information and responses would be confidential and anonymous. A total of 1,100 questionnaires were distributed to kindergarten teachers in Henan, Anhui, and Xinjiang, China; 1,030 questionnaires were actually returned, with a response rate of 93.6%. Of these 1,030 questionnaires, 1,014 were valid, with an effectivity rate of 92.2%. These 1,014 valid questionnaires formed the final sample of this study. The demographic characteristics of the samples are shown in Table 1.

**Table 1:** Demographic information of kindergarten teacher sample (*n* = 1,014).

Characteristics	*n* (%)
**Area**	
Urban	690 (68.0)
Rural	324 (32.0)
**Education level**	
≤High school	96 (9.5)
Vocational school	429 (42.3)
≥University degree	489 (48.2)
**Teaching experience**	
≤5 years	614 (60.6)
6–10 years	220 (21.7)
11–15 years	84 (8.3)
16–20 years	39 (3.8)
≥21 years	57 (5.6)

### Measures

#### Total Rewards Perceptions for Kindergarten Teacher

The Total Rewards Perceptions Scale for Kindergarten Teachers was adapted from the four-factor total rewards perceptions scale that was designed by Chinese researcher Yang and Yang (2015), which was validated on the basis of a total rewards model that includes compensation, benefits, work–life balance, performance and recognition, and development and career opportunities proposed by the WorldatWork (2007). The scale, consisting of 21 items, was used to measure four dimensions of total rewards perceptions: work–life balance perceptions (WLBP; e.g., kindergarten guarantees teachers' rest time), development and career opportunity perceptions (DCOP; e.g., kindergarten provides teachers with a clear path to future advancement), working conditions perceptions (WCP; e.g., kindergarten pays five social insurance and one housing fund for teachers in full and on time), and wage level perceptions (WLP; e.g., the wage level of the kindergarten matches with my professional skills). The 21 items were measured using a five-point Likert-type scale, ranging from 1 (very poor) to 5 (very well). The higher the score on the four dimensions, the better the total rewards perceived by kindergarten teachers. In this study, Cronbach's α was 0.96. Internal consistency was 0.89 for WLBP, 0.92 for DCOP, 0.85 for WCP, and 0.92 for WLP.

#### Kindergarten Teacher Work Engagement

Kindergarten Teacher Work Engagement was measured using the Utrecht work engagement scale (UWES) developed by Schaufeli et al. (2002). The scale has been used in different samples of Chinese and shows good reliability and validity (Wang et al., 2015; Li and Wu, 2016). This scale included three dimensions: vigor (e.g., I can continue working for very long periods at a time), dedication (e.g., I am proud of the work that I do), and absorption (e.g., I get carried away when I am working). The responses for all of the items were obtained on a five-point Likert scale (1 = strongly disapprove, 5 = strongly approve). In this study, Cronbach's α was 0.96. Internal consistency was 0.89 for vigor, 0.92 for dedication, and 0.90 for absorption.

#### Kindergarten Teacher Organizational Identification

Kindergarten Teacher Organizational Identification Scale was adopted from the Organizational Identification Scale developed by Mael and Ashforth (Mael and Ashforth, 1992). This scale has illustrated good reliability and validity in Chinese samples (Wang et al., 2016; Song et al., 2019). The scale consisted of six items, which were scored on a five-point Likert scale from 1 (strongly disagree) to 5 (strongly agree). A sample item was “I think that the success of my kindergarten is also my success.” In this study, Cronbach's α was 0.86.

#### Control Variables

We controlled the potential effect of demographic variables (area, education level, and teaching experience) on dependent and mediating variables, as all of them may impact employee work engagement.

### Procedure

This project was reviewed and approved by the Ethics Committee of Huaibei Normal University and complied with the Declaration of Helsinki involving human subjects. We obtain consent from the kindergarten principal to enter the kindergarten to distribute questionnaires in 2019. Before the test, participants would be given informed consent and were informed about the research content and their rights. We also informed participants that completion of the test was entirely voluntary and that they had the right to decline to complete the test. The test would continue after participants confirm informed consent. If participants refused to participate, the test ended.

### Data Analysis

In the current study, we used SPSS 22 software to analyze the relationship between total rewards perceptions, organizational identification, and work engagement among kindergarten teachers. The descriptive statistics (mean and standard deviation) were calculated to measure the level of all variables. Bivariate correlations were used to examine correlations between socio-anagraphical variables, kindergarten teachers' total rewards perceptions (including WLBP, DCOP, WCP, and WLP), organizational identification, and work engagement. We used structural equation modeling (SEM) in Mplus 8.0 to investigate the impact of organizational identification on the relationship between total rewards perceptions and work engagement. The comparative fit index (CFI), the Tucker–Lewis index (TLI), the root mean square error approximation (RMSEA), and the standardized root mean square residual (SRMR) were used to estimate the model fit information. Hu and Bentler (1999) suggested that TLI, CFI > 0.90, and RMSEA, SRMR < 0.08 showed the model fitted well. The bootstrapping method of bias correction was used to verify the significance of the mediation effect. No zero between the lower level and higher levels of the confidence interval means that a mediating effect is significant (Hayes, 2009).

## Results

### Descriptive Statistics and Correlations Among the Variables

Table 2 presents the descriptive statistics and correlations among variables. Among the four factors of total rewards perceptions, WCP (M = 3.92, SD = 0.84) scored higher than WLBP (M = 3.75, SD = 0.86), DCOP (M = 3.79, SD = 0.79), and WLP (M = 3.78, SD = 0.82). The mean score of organizational identification and work engagement were 4.14 (SD = 0.72) and 3.94 (SD = 0.73). Kindergarten teachers' total rewards perceptions were positively associated with work engagement (*r* = 0.62, *p* < 0.01) and organizational identification (*r* = 0.30, *p* < 0.01). Four factors of total rewards perceptions were positively related to work engagement (WLBP, *r* = 0.59, *p* < 0.01; DCOP, *r* = 0.61, *p* < 0.01; WCP, *r* = 0.40, *p* < 0.01; WLP, *r* = 0.58, *p* < 0.01) and organizational identification (WLBP, *r* = 0.22, *p* < 0.01; DCOP, *r* = 0.26, *p* < 0.01; WCP, *r* = 0.28, *p* < 0.01; WLP, *r* = 0.28, *p* < 0.01). The correlation among DCOP and work engagement was higher than that among the other three factors of total rewards perceptions and work engagement. Organizational identification correlated positively with work engagement (*r* = 0.41, *p* < 0.01). H1 was supported. In addition, education level and teaching experience are correlated with TRP, OI, and WE to different degrees. Therefore, education level and teaching experience were controlled in subsequent analysis.

**Table 2:** Descriptive statistics and correlations among variables.

**Variable**	**1**	**2**	**3**	**4**	**5**	**6**	**7**	**8**	**9**	**10**
1. Area	—									
2. EL	0.12^**^	—								
3. TE	−0.94^**^	0.19^**^	—							
4. TRP	0.038	0.053	−0.08^*^	—						
5. WLBP	−0.03	−0.14^**^	0.07^*^	0.87^**^	—					
6. DCOP	−0.02	−0.14^**^	0.04	0.92^**^	0.79^**^	—				
7. WCP	0.14^**^	0.07^*^	0.07^*^	0.83^**^	0.56^**^	0.66^**^	—			
8. WLP	0.04	−0.08^**^	−0.00	0.91^**^	0.70^**^	0.80^**^	0.72^**^	—		
9. OI	0.03	0.10^**^	0.14^**^	0.30^**^	0.22^**^	0.26^**^	0.28^**^	0.28^**^	—	
10. WE	−0.03	−0.08^**^	0.17^**^	0.62^**^	0.59^**^	0.61^**^	0.40^**^	0.58^**^	0.41^**^	—
M	—	—	1.72	3.81	3.75	3.79	3.92	3.78	4.14	3.94
SD	—	—	1.13	0.73	0.86	0.79	0.84	0.82	0.72	0.73

*n = 1,014;*

* *p < 0.05*,

** *p < 0.01 (double tails)*.

*EL, education level; TE, teaching experience; TRP, total rewards perceptions; WLBP, work–life balance perceptions; DCOP, development and career opportunity perceptions; WCP, working conditions perceptions; WLP, wage level perceptions; OI, organizational identification; WE, work engagement*.

### Mediating Effect

**Figure 2:**
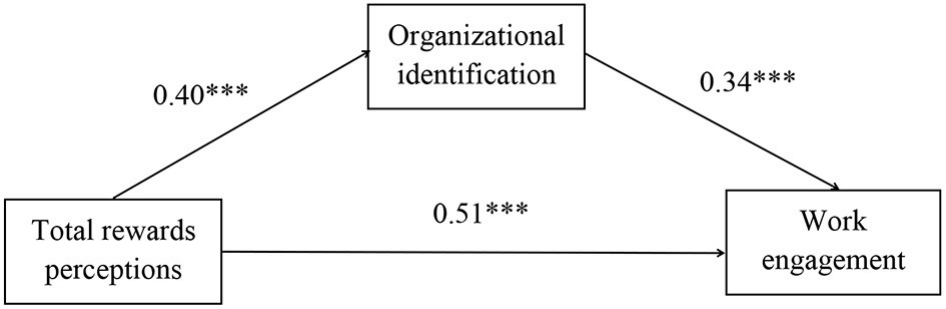
The SEM analysis conducted to examine the pathways among total rewards perceptions, organizational identification, and work engagement. The control variables are not included in the presentation of the model. ***p < 0.001.

A standardized structural equation model was adopted to investigate the mediation effect of organizational identification in the relationship between total rewards perceptions and work engagement. We control the demographic variables (education level and teaching experience) in the structural equation model. The model consisted of total rewards perceptions, organizational identification, and work engagement. The results showed this model had good fitting indices: χ^2^ = 184.47 (*p* < 0.001), df = 67, χ^2^/df = 2.75, CFI = 0.96, TLI = 0.95, RMSEA = 0.06 [90% CI = (0.05, 0.07)], SRMR = 0.04. Total rewards perceptions positively predicted organizational identification (β = 0.40, *p* < 0.001). Organizational identification (β = 0.34, *p* < 0.001) significantly positively predicted work engagement. The direct effect of total rewards perceptions on work engagement was significant (β = 0.51, *p* < 0.001). Hence, the results of the indirect effects demonstrated that organizational identification (β = 0.14, *p* < 0.001) mediated the correlation between total rewards perceptions and work engagement. Furthermore, bootstrapping test showed that the mediation effect of organizational identification was significant [95% CI = (0.121, 0.230)] (see Table 3 and Figure 2). Thus, H2 was supported.

**Table 3:** Mediating effect of organizational identification.

	**β**	***p***	**LLCI**	**ULCI**
TRP → WE (c)	0.51	<0.001	0.436	0.570
TRP → OI (a)	0.40	<0.001	0.332	0.472
OI → WE (b)	0.34	<0.001	0.269	0.422
TRP → OI → WE	0.14	<0.001	0.098	0.180

*c, the direct effect of total rewards perceptions on work engagement; a, the effect of total rewards perceptions on organizational identification; b, the effect of organizational identification on work engagement; TRP, total rewards perceptions; OI, organizational identification; WE, work engagement*.

## Discussion

The present study examined the correlation among total rewards perceptions, organizational identification, and work engagement among Chinese kindergarten teachers. Past studies have explored the correlation between total rewards perceptions and work engagement, or organizational identification and work engagement, but have not presented the correlation among the three variables. This study found that WLBP, DCOP, WCP, and WLP correlated significantly and positively with work engagement. The obtained results corroborate the finding of a previous study (Gulyani and Sharma, 2018), which indicated that a high level of monetary rewards and non-monetary rewards may foster employee engagement in work. Employees who were offered a reward package that is consistent with their personal preferences were more likely to engage in work (Pregnolato et al., 2017). Bakker et al. (2014) indicated that work resources such as training, development opportunities, and additional benefits can motivate employees to involve themselves in work.

This study found that DCOP of total rewards perceptions had a stronger positive relationship with work engagement than WLBP, WCP, and WLP. It was consistent with existing research (Scott and McMullen, 2010), which reported that quality of work career development, organizational culture, and work–life balance all have a greater impact on work engagement than pay. Hulkko-Nyman et al. (2012) estimated that non-monetary rewards have a greater correlation with work engagement than monetary rewards. Kindergarten is perceived as a low-wage unit in China. The level of financial investment in kindergartens in China is much lower than that in OECD countries (Liu and Huang, 2019). The low monetary rewards are considered normal among kindergarten teachers. Moreover, teaching is a helping profession, with a great component of idealist motivation (Xu and Du, 2014). Thus, the impact of monetary rewards on work engagement is not as strong as the impact of non-monetary rewards on work engagement among kindergarten teachers.

This study also found that kindergarten teachers' total rewards perceptions were positively related to organizational identification. The finding was also in agreement with a previous study (Yang and Yang, 2015). Past research has shown that employees develop a positive attitude toward the workplace when they have access to rewards such as benefits, career development opportunities (Abid et al., 2015), and pay rise (Oishi et al., 2011). Other studies have stated that total rewards have a significant positive impact on organizational support, which was closely related to organizational identification (Smit et al., 2015; Zagenczyk et al., 2020). In China's cultural context, people have a strong concept of collectivism. Collectivists are more likely to see themselves as part of the organization, show more attachment to the organization, take pride in their membership, and have good organizational identification in a collectivist climate (Hofstede, 2003; Roth et al., 2011). Therefore, the influence of kindergarten teachers' total rewards perceptions on organizational identification should also be considered in the cultural context of Chinese collectivism.

We also found that the influence of WCP and WLP is greater than that of WLBP and DCOP. This result contradicts the finding from previous research in the Western context (Goulet and Frank, 2002; Steijn and Leisink, 2006), which stated that monetary rewards have limited impact on employees' organizational commitment. However, the result is consistent with research on Chinese organizations (Chiu et al., 2002; Miao et al., 2013), which found that monetary rewards (e.g., pay, fringe benefits) are the main factors that affect organizational commitment.

This study suggested that organizational identification was positively correlated with teachers' work engagement. The finding was in accordance with previous studies (Zhang et al., 2018, 2020; Li et al., 2020), which indicated that the greater organizational identification, the higher level of employee engagement in the work. Organizational identification enables employees to internalize the success of the organization into their success; thus, it has a positive impact on work engagement (Karanika-Murray et al., 2015).

Rewards from the Kindergarten can motivate teachers to focus and engage at work, while teachers' attitudes toward work engagement vary from context to context. Kindergarten teachers have different needs and expectations in terms of rewards. For instance, some employees expect material rewards in return for their hard work, while others expect return in the form of non-monetary rewards (Nazir et al., 2016). Therefore, examining the total rewards will help identify the combinations of rewards that motivate Kindergarten teachers to engage in work.

The present study found that organizational identification partially mediated the relationship between total rewards perceptions and work engagement among kindergarten teachers, which fit the Social Exchange Theory. This theory takes the exchange between organization and employees as the core (Blau, 1964). Organizations provide employees with material and emotional support, employees identify with the organization, and the exchange relationship arises (Blau, 1964; Emerson, 1976). The quality and sustainability of the exchange are affected by the rewards obtained by both parties through the exchange (Homans, 1958). When employees are rewarded for their hard work, they participate in an exchange that increases their work engagement for organization (Gujral and Jain, 2013). Some empirical studies indicated that offering rewards from organization to employees implies that the organization expects to engage in a social exchange with them, and an influential psychological contract is established between organization and employees (Williamson et al., 2009; Newman and Sheikh, 2012b). Meanwhile, Social Identity Theory states that the strong relationship between employees and the organization may motivate employees to give their best for the organization (Brown, 2017; Pan et al., 2019) and increase their level of engagement (Wang and Tseng, 2019). When kindergarten teachers get the expected economic or non-economic rewards from the organization, in return, they will have a stronger sense of identity with kindergarten, form a positive psychological state, and immerse themselves in their work (Blau, 1986; Gulyani and Sharma, 2018). The Conservation of Resources Theory proposes that individuals with more resources not only will try their best to maintain and protect their existing resources but also are more capable of acquiring new resources (Hobfoll, 1989, 2011), thus exhibiting more positive mental states and behaviors (Halbesleben and Wheeler, 2008). Gorgievski and Hobfoll (2008) also indicated that people with ample resources are more likely to approach their work with energy and enthusiasm; thus, they have higher levels of engagement in work. The total rewards system, as an effective job resources program, may achieve the goal of creating positive feelings among teachers (Bakker et al., 2014) and affect employee organizational identification (Hwang and Jang, 2020). Individuals with high levels of organizational identification are more likely to enhance their work engagement (Riketta, 2005). Therefore, kindergarten teachers' total rewards perceptions not only influence work engagement directly but also indirectly affect work engagement by increasing their organizational identification.

### Limitations and Future Research Directions

The present study has several limitations that need to be emphasized. First, the data for this study were collected from three provinces in China, which lack diverse national settings. Future studies could expand the scope of data collection as far as possible. Second, a cross-sectional research design was used in this study; thus, it cannot predict to what extent changes in kindergarten teachers' total rewards perceptions and organizational identification will lead to changes in work engagement. A longitudinal approach should be used to validate the causal relationship between kindergarten teachers' total rewards perceptions, organizational identification, and work engagement in future studies. Third, we used the Social Exchange Theory and Conservation of Resources Theory to explain the relationship among study variables and mediating effect of organizational identification, which have certain limitations, and other theoretical perspectives should be further explored. Finally, the mediating effect of organizational identity was small, suggesting that other mediators could explain the relationship between teachers' total rewards perceptions and work engagement. More mediating variables should be examined among total rewards perceptions, organizational identification, and work engagement relationship.

### Conclusions

This study contributes to enriching the existing research literature and finds that except for the direct relationship, the relationship between total rewards perceptions and work engagement was partially mediated by organizational identification among Chinese kindergarten teachers. We use Social Exchange Theory and Conservation of Resources Theory to explain the mediating role of organizational identification. Other possible mediators between total rewards perceptions and work engagement and other antecedent variables of work engagement deserve more in-depth research and exploration.

## Data Availability Statement

The raw data supporting the conclusions of this article will be made available by the authors, without undue reservation.

## Ethics Statement

The studies involving human participants were reviewed and approved by The Ethics Committee of College of Education, Huaibei Normal University. The patients/participants provided their written informed consent to participate in this study.

## Author Contributions

DJ and LC designed the study, reviewed, and revised the article together. DJ wrote the original draft of the manuscript. LC collected and analyzed the survey data. All authors contributed to the article and approved the submitted version.

## Conflict of Interest

The authors declare that the research was conducted in the absence of any commercial or financial relationships that could be construed as a potential conflict of interest.
